# Orthodontic treatment in periodontitis‐susceptible subjects: a systematic literature review

**DOI:** 10.1002/cre2.28

**Published:** 2016-04-21

**Authors:** Egle Zasciurinskiene, Rune Lindsten, Christer Slotte, Krister Bjerklin

**Affiliations:** ^1^ Department of Orthodontics, Medical Academy Lithuanian University of Health Sciences Kaunas Lithuania; ^2^ School of Health Sciences Jönköping University Jönköping Sweden; ^3^ Department of Orthodontics Institute for Postgraduate Dental Education Jönköping Sweden; ^4^ Department of Periodontology Institute for Postgraduate Dental Education Jönköping Sweden; ^5^ Department of Biomaterials, Institute for Clinical Sciences, Sahlgrenska Academy University of Gothenburg Göteborg Sweden

**Keywords:** Alveolar bone loss, chronic periodontitis, humans, orthodontic tooth movement, periodontal pocket depth, periodontal treatment

## Abstract

The aim is to evaluate the literature for clinical scientific data on possible effects of orthodontic treatment on periodontal status in periodontitis‐susceptible subjects. A systematic literature review was performed on studies in English using PubMed, MEDLINE, and Cochrane Library central databases (1965‐2014). By manually searching reference lists of selected studies, we identified additional articles; then we searched these publications: *Journal of Periodontology*, *Periodontology 2000*, *Journal of Clinical Periodontology*, *American Journal of Orthodontics and Dentofacial Orthopedics*, *Angle Orthodontist*, *International Journal of Periodontics & Restorative Dentistry*, and *European Journal of Orthodontics*. Search terms included randomized clinical trials, controlled clinical trials, prospective and retrospective clinical studies, case series >5 patients, periodontitis, orthodontics, alveolar bone loss, tooth migration, tooth movement, orthodontic extrusion, and orthodontic intrusion. Only studies on orthodontic treatment in periodontally compromised dentitions were included. One randomized controlled clinical trial, one controlled clinical trial, and 12 clinical studies were included. No evidence currently exists from controlled studies and randomized controlled clinical trials, which shows that orthodontic treatment improves or aggravates the status of periodontally compromised dentitions.

## Introduction

Periodontitis is a polymicrobial infection that results in a destructive host response to the supporting apparatus of the dentition (Nishihara and Koseki, 2004). General, behavioral, genetic, and environmental risk factors (e.g., smoking) modify the immunoinflammatory response, which results in more severe periodontal destruction (Page and Kornman, 1997; Kornman, 2008). Local risk factors are associated with worsened prognosis of periodontally involved teeth (Hallmon, 1999; Harrel, 2003; Harrel *et al*., 2006; Harrel and Nunn, 2009).

Chronic periodontitis treatment is complex. Despite new modifications in recent years, supragingival and subgingival deposit and bacterial biofilm removal (through scaling and root planing) are the gold standard of chronic periodontitis treatment (Sanz *et al*., 2012; Plessas, 2014) that follows the mandatory supragingival plaque control.

Periodontal complications and posterior tooth loss may lead to posterior‐occlusion collapse and vertical‐dimension reduction – often causing proclination, spacing, and over‐eruption of anterior teeth. Changes in tooth position may complicate plaque control, traumatize periodontium, and lead to unsatisfactory esthetics and function (Johal and Ide, 1999). Research supporting occlusal interventions as adjunctive treatment of periodontitis in adults is scarce and leads to the conclusion that no evidence is present for or against use of occlusal interventions in clinical practice (Weston *et al*., 2008).

To test effects of orthodontic tooth movement on reduced periodontium, several experimental animal studies were published. Ericsson *et al*. (1977, 1978) studied orthodontic tooth movement in dogs and concluded that healthy and inflamed periodontal tissues react differently. Movement of teeth – when having reduced but healthy periodontium – did not cause additional attachment loss (Ericsson *et al*., 1978). Also, mesial movement into infrabony defects in rats (Vardimon *et al*., 2001; Nemcovsky *et al*., 2004, 2007) and intrusion movement in monkeys (Melsen *et al*., 1988; Melsen, 2001), and extrusion movement in dogs (van Venrooy and Yukna, 1985), were performed without additional loss of periodontal support, provided oral hygiene was maintained. On the contrary, orthodontic movement of teeth with inflamed infrabony pockets was found to increase loss of connective tissue attachment (Ericsson *et al*., 1977; Wennstrom *et al*., 1987; Melsen, 2001).

Findings in animal studies with experimentally induced periodontal disease cannot be easily extrapolated to human conditions because natural periodontal destruction is unknown in monkeys and it occurs in much older dogs than those used in the studies. Attachment loss in humans occurs relatively slowly over a much longer time (Harrel *et al*., 2006), and underlying modifying host responses possibly influence it. Hence, orthodontic treatment of occlusal discrepancies in chronic periodontal disease cases remains controversial.

The aim of this systematic literature review was to identify data on possible effects of orthodontic treatment on periodontal status in periodontitis‐susceptible subjects.

*Null hypothesis*: no evidence‐based studies are available on the effect of orthodontic therapy on patients with a history of chronic periodontitis.

## Material and methods

We systematically reviewed the literature, based on the PRISMA statement (Liberati *et al*., 2009), and developed a protocol to describe the population, intervention, comparison, and outcomes format (Richardson *et al*., 1995). *Types of participants*: Only studies on treatment of adult patients with a periodontal disease history were included. *Types of intervention*: We limited the review to studies that assess changes in periodontal tissues when periodontal and orthodontic treatment was administered in patients with periodontitis. *Comparison*: Periodontal tissue reactions in periodontally susceptible subjects, who received various orthodontic interventions, were compared with periodontally healthy subjects. *Outcome measures*: Changes in periodontal pocket depth (PPD), clinical crown height (CCH), bleeding on probing, alveolar bone level, and root resorption.

### Literature search strategy

We conducted a detailed search (the 1965–June 2014 period) using the PubMed, MEDLINE, and Cochrane Library central databases. In addition, these journals were searched: *Journal of Periodontology*, *Periodontology 2000*, *Journal of Clinical Periodontology*, *American Journal of Orthodontics and Dentofacial Orthopedics*, *Angle Orthodontist*, *International Journal of Periodontics* & *Restorative Dentistry*, and *European Journal of Orthodontics*. A librarian at the Lithuanian University of Health Sciences assisted in developing a search strategy.

*Eligibility criteria*: Table 1 lists predefined inclusion and exclusion criteria. *Search string*: Tables 2 and 3 show the search strategy for the PubMed and MEDLINE search engines with medical subheadings. *Manual searching*: Reference lists of selected articles were studied.

**Table 1:** Systematic literature review analysis inclusion criteria for clinical studies.

Inclusion criteria	Exclusion criteria
Randomized controlled clinical trials Prospective controlled clinical trials Prospective cohort studies Retrospective cohort studies Case series >5 patients English language	Case reports or series ≤5 patients Animal studies Review papers and abstracts Reports Letters to the editor Conference abstracts Articles with no periodontal disease history Articles with aggressive periodontitis cases

**Table 2:** Search words and phrases and number of articles found.

	Search words and phrases	No. of articles
1	(“periodontitis”[MeSH]) AND “orthodontics”[MeSH]	447
2	(“alveolar bone loss”[MeSH]) AND “orthodontics, corrective”[MeSH]	303
3	(“tooth migration”[MeSH]) AND “orthodontics, corrective”[MeSH]	322
4	(“tooth movement”[MeSH]) AND “alveolar bone loss”[MeSH]	150
5	“alveolar bone loss”[MeSH] AND (“orthodontic extrusion”[MeSH Terms]) OR (“orthodontic”[All Fields] AND “extrusion”[All Fields]) OR (“orthodontic extrusion”[All Fields])	29
6	“alveolar bone loss ”[MeSH] AND (“orthodontic intrusion”[MeSH Terms]) OR (“orthodontic”[All Fields] AND “intrusion”[All Fields]) OR (“orthodontic intrusion”[All Fields])	12
7	1 AND 2	80
8	2 AND 3	18

**Table 3:** Search string for journals in MEDLINE and number of articles found.

	String used in MEDLINE	No. of articles
1	“J Periodontol”[Journal] AND orthodontics AND periodontitis	29
2	“J Periodontol”[Journal] AND orthodontics AND alveolar bone loss	27
3	“J Clin Periodontol”[Journal] AND orthodontics AND periodontitis	21
4	“J Clin Periodontol”[Journal] AND orthodontics AND alveolar bone loss	12
5	“Am J Orthod Dentofacial Orthop”[Journal] AND orthodontics AND periodontitis	43
6	“Am J Orthod Dentofacial Orthop”[Journal] AND orthodontics AND alveolar bone loss	87
7	“Angle Orthod”[Journal] AND orthodontics AND periodontitis	20
8	“Angle Orthod”[Journal] AND orthodontics AND alveolar bone loss	28
9	“Int J Periodontics Restorative Dent”[Journal] AND orthodontics AND periodontitis	15
10	“Int J Periodontics Restorative Dent”[Journal] AND orthodontics AND alveolar bone loss	18
11	“Eur J Orthod”[Journal] AND orthodontics AND periodontitis	12
12	“Eur J Orthod”[Journal] AND orthodontics AND alveolar bone loss	21
13	“Periodontol 2000”[Journal] AND orthodontics	13

### Screening and selection

Reading study titles enabled attainment of the initial number of identified records (via electronic searches). Three authors of the present review (E. Zasciurinskiene, R. Lindsten, and C. Slotte) independently selected titles to obtain the studies' abstracts. As per inclusion criteria, they independently assessed eligibility of selected abstracts in an unblinded manner. Studies were excluded using eligibility criteria, namely, researchers' conclusions and type of study, participants, intervention, and outcome. Full texts of relevant studies were retrieved.

### Data extraction

Three authors of the present review (E. Zasciurinskiene, R. Lindsten, and C. Slotte) performed data extraction. These characteristics of included studies were identified for reporting; see Table 4.

1. General characteristics: year of study.
2. Population studied: adults with chronic periodontal disease.
3. Study design: sample size, teeth tested, and presence of periodontally healthy controls. For random clinical trials, allocation method, allocation concealment, blinding, and comparative group characteristics.
4. Character of intervention, that is, type of periodontal intervention, orthodontic appliances, and movements.
5. Outcomes measured: change in CCH, mean probing depth change, proximal bone level change, and adverse effects such as root resorption.
6. Clinical conclusions.

**Table 4:** Summarized data of the 14 studies that fulfilled inclusion criteria.

Reference	Study design	Participants (test patients and teeth)	Type of orthodontic appliances and movement	Periodontal intervention, before orthodontic treatment	Change in clinical crown height	Change in mean PPD before–after orthodontic treatment	Proximal bone level changes before–after orthodontic treatment	Root resorption	Clinical conclusion of orthodontic tooth movement impact on periodontall involved teeth
Eliasson *et al*., 1982	Clinical observational	20 adults Test: 71 maxillary incisors Controls: 40 not moved canines or first premolars	Removable appliances Tipping movement	Supragingival and subgingival scaling 4–6 months before Perio‐surgery after	NM^a^	NC^a^	NC in 52% Reduced in 30% Improved in 18%	No root resorption	No significant loss of attachment occurred; did not affect periodontal tissue status
Artun and Urbye, 1988	Retrospective clinical	24 adults Test: maxillary anterior teeth Controls: not moved teeth in opposite jaw	Fixed appliances Bodily movement	Scaling, root planing before Surgery after	NM	NM	Bone loss In test teeth 4.94% In control teeth 2.69% Bone gain 7 sites in test 13 sites in controls	20 teeth in 11 patients	Loss of periodontal bone support may occur.
Boyd *et al*., 1989	Comparative clinical	10 perio adults 10 non‐perio adults Test: All teeth Controls: All teeth of 20 adolescents	Fixed appliances Bodily movement	Root planing before Flap surgery before	NM	ND^a^	NM	NM	No loss of attachment, if reduced but healthy periodontium; not healthy periodontally involved teeth may experience periodontal breakdown and tooth loss
Melsen *et al*., 1989	Clinical	30 adults Test: Maxillary anterior teeth No controls	Four types of fixed appliances Intrusion	Curettage before 50% needed surgery 1 week before 5 patient needed surgery after	1.08 mm reduction	PPD increase of about 3 mm on lingual surfaces	Unaltered or increased in 19 from 30 cases	All cases had root resorption 1–3 mm	In most cases, beneficial effect on periodontal condition at clinical and radiographic level
Burch *et al*., 1992	Retrospective clinical	16 adults Test: 20 mandibular molars No controls	Limited fixed appliances Uprighting	Not described	NM	Increased in 35%, decreased in 7.5%, no change in 57.5%	60% mesial bone loss	NM	About 50% of furcation areas became more severe; loss of attachment mesially due to extrusion of mesial root. Light forces with intrusive component are recommended.
Re *et al*., 2000	Retrospective clinical	267 adults Test: maxillary anterior teeth‐surgery group Controls: maxillary anterior teeth‐non surgery group	Fixed appliances Intrusion	129 perio‐surgery 128 non surgical 1 week before	NM	Reduced 2.97 ± 0.78 mm ND between groups.	NM	NM	Combination of orthodontic intrusion and periodontal treatment improved condition with reduced periodontal support.
Melsen, 2001	Prospective clinical	30 adults Test: 4 maxillary incisors No controls	Fixed appliances Intrusion with proclination or retroclination	Widman flap surgery before	Reduced in 28 subjects	NM	Increased in 25 subjects	NM	Tissue reaction depended on perio‐status of the teeth. Intrusion improved perio‐status of healthy periodontium.
Cardaropoli *et al*., 2001, b	Clinical	10 adults Test: Maxillary incisors No controls	Fixed appliances Intrusion	Open flap surgery 7–10 days before	Reduction 1.05 ± 0.5 mm	Reduced by 4.35 ± 0.42 mm	Reduction of bone defect by 4.36 mm	No root resorption	Intrusion of maxillary incisors after surgery may be a reliable method in patients with extrusion and the presence of angular bony defect.
Corrente *et al*., 2003, b	Clinical	10 adults Test: maxillary incisors No controls	Fixed appliances Intrusion	Open flap surgery 7–10 days before	CAL gain 5.50 ± 1.75 mm	Reduction of 4.35 ± 1.33 mm	Bone fill of 1.35 ± 0.75 mm vertically 1.40 ± 0.88 mm horizontally	No root resorption	Combined orthodontic–periodontic treatment resulted in radiological bone fill, CAL gain, PPD, and recession reduction.
Re *et al*., 2004, c	Clinical	28 adults Test: maxillary incisors No controls	Fixed appliances Intrusion	Open flap surgery 7–10 days before	Reduction by 1.71 mm mesially 0.96 mm buccally	Mesial PPD reduced by 4.29 mm	NM	NM	Positive outcome of parameters examined
Cardaropoli *et al*., 2004 c	Clinical	28 adults Test: maxillary incisors No controls	Fixed appliances Intrusion	Open flap surgery 7–10 days before	Reduction by 1.71 mm mesially 0.96 mm buccally	Reduced by 4.29 mm	NM	NM	Midline papilla reconstruction was positive in 82% of treated patients and favored esthetics.
Ghezzi *et al*., 2008	Comparative clinical	14 adults Test: maxillary incisors No controls	Fixed appliances Intrusion and bodily movement	GTR procedure EMD for three‐wall defects. Bone graft for one‐wall and/or two‐wall defect 1 year before	NM	PPD reduction by 5.57 mm, 1 year after GTR. Additionally reduced by 0 .07 mm after orthodontic treatment	NM	NM	General improvement of PPD, CAL, and esthetic parameters occurred. Papilla enhancement in nine of 14 patients.
Ogihara and Wang, 2010	Randomized parallel clinical trial	47 adults Test: ortho tx/EMD/DFDBA (*n* = 24) Controls: EMD/DFDBA (*n* = 23)	Segmentally fixed appliances Extrusion	Perio‐surgery + EMD/DFDBA 4 weeks before	NM	Both groups PPD reduction	Gain in both groups for the two‐wall defect sites	NM	Both treatments EMD/DFDBA and Ortho/EMD/DFDBA were effective.
Attia *et al*., 2012	Controlled clinical trial	I gr‐5‐ortho tx immediately after surgery; II gr‐5‐ortho tx 2 months after surgery Controls: 5 – surgery without ortho tx	Segmented arch technique Towards bony defect	Scaling root planing before + flap surgery filled with bio‐glass and collagen membrane before	ND	PPD reduction all groups	Significantly increased in all groups	NM	Combined orthodontic regenerative therapy resulted in favorable clinical and radiographic outcomes

Ortho tx, orthodontic treatment; EMD, enamel matrix derivative; DFDBA, demineralized freeze‐dried bone allograft.

a NM, not measured; ND, no difference; NC, no change.

b These two studies used the same patient material.

c These two studies used the same patient material.

### Quality assessment

#### Newcastle–Ottawa quality assessment scale

Two authors (Egle Zasciurinskiene and Rune Lindsten) assessed the methodological quality of selected articles using a Newcastle–Ottawa scale for case–control and cohort studies (Wells *et al*., 2001). After filling in each score sheet, they provided a total assessment of the quality of the reviewed article. The *star* system was applied to each study; it is based on these items (Table 5):

- Selection (i.e., study groups that represented periodontal disease parameters and control groups without periodontally involved adults): maximum of four stars.
- Comparability (comparability of cases and controls as per the study design or analysis): maximum of two stars.
- Exposure of interest (i.e., changes in periodontal parameters): maximum of three stars.
- Statistical analysis (statistical analysis and unit of analysis validities): maximum of two stars.

**Table 5:** The methodological quality evaluation of included studies.

Reference	Selection	Comparability	Exposure	Statistics	Sum
Eliasson *et al*., 1982	**	–	*	**	5
Artun and Urbye, 1988	–	–	*	*	2
Boyd *et al*., 1989	**	*	*	*	5
Melsen *et al*., 1989	*	–	*	–	2
Burch *et al*., 1992	**	–	*	–	3
Re *et al*., 2000	**	–	*	*	5
Melsen, 2001	*	–	*	–	2
Cardaropoli *et al*., 2001	**	–	*	*	4
Corrente *et al*., 2003	**	–	*	*	4
Re *et al*., 2004	**	–	*	**	5
Cardaropoli *et al*., 2004	**	–	*	**	5
Ghezzi *et al*., 2008	**	–	*	*	4
Attia *et al*., 2012	**	–	*	*	4

Studies with 9–11 stars were considered to have high methodological quality; 6–8 stars, medium quality; and less than six stars, low quality. Methodological quality for randomized controlled clinical trials (RCTs) was assessed as described in the *Cochrane Handbook for Systematic Reviews of Interventions* (Higgins and Green, 2011).

To reach consensus, any conflicts among the authors were resolved via discussion of each study.

## Results

The PubMed and MEDLINE searches yielded 1361 article titles; 346 article titles appeared in MEDLINE‐indexed journals. From 113 articles found in the Cochrane Library central database, only 12 matched inclusion criteria. Two articles of the 12 were identified for inclusion in the review (these also came up in the PubMed–MEDLINE search).

**Figure 1:**
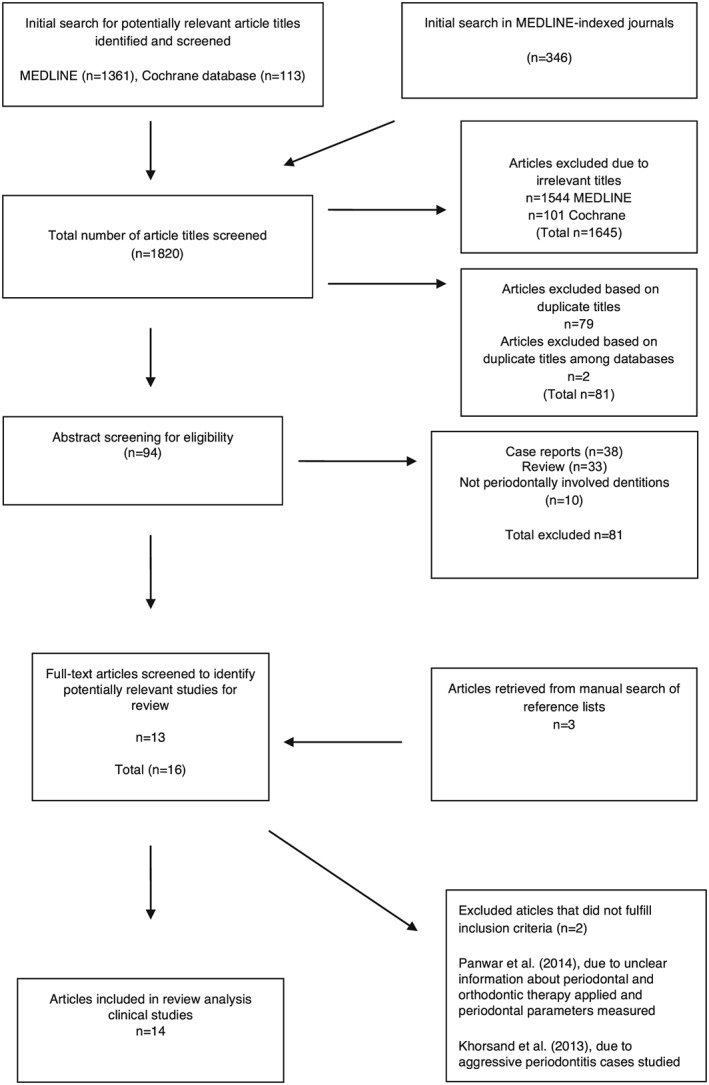
Search‐and‐analysis process and results (*n*) from each stage.

Figure 1 illustrates the search process. The search strategy resulted in 1820 article titles. After combining the aforementioned results with medical subheadings results (from screening titles and removing duplicates), we excluded 1726 titles and selected 94 studies for further evaluation. When evaluating abstracts of the selected 94 studies (as per inclusion and exclusion criteria), the aforementioned reviewers determined that 13 studies (plus three additional manually searched studies, identified via manual searches of reference lists in selected articles) were relevant for the present review.

In total, 81 studies were excluded (as per exclusion criteria) after screening the abstracts. Figure 1 contains reasons for exclusion. Full texts of the 16 relevant studies were retrieved. After reading these 16 articles, the Panwar *et al*. (2014) study was excluded because of (i) unclear information about applied periodontal and orthodontic therapies and (ii) lack of relevant, measured periodontal parameters. After discussion among the aforementioned authors, the Khorsand *et al*. (2013) study was also excluded because only aggressive periodontitis cases were studied. Consequently, 14 full‐text articles were included for final evaluation; see Table 4.

### Reviewed studies' designs and treatment methods

Only one study was an RCT (Ogihara and Wang, 2010), and one was a controlled clinical trial (Attia *et al*., 2012). The RCT investigated the effect of segmented orthodontics, combined with reconstructive surgery, on premolar or molar teeth with two‐wall or three‐wall infrabony defects. The controlled clinical trial studied effectiveness of different timing for initiating active orthodontic treatment after surgical reconstructive procedures, when treating infrabony defects. Nine studies were prospective studies, and three were retrospective clinical studies.

All 14 studies investigated periodontal changes during orthodontic treatment in periodontally compromised dentitions; Table 4 summarizes these studies.

In 10 clinical studies, periodontal surgery was performed before orthodontic treatment (three of these studies used guided tissue regeneration). Eliasson *et al*. (1982) and Artun and Urbye (1988), however, performed corrective periodontal surgery after orthodontic treatment. The molar uprighting study (Burch *et al*., 1992) did not describe periodontal treatment. The Attia *et al*. (2012) study reported that no significant difference occurred in PPD reduction among groups that were assigned various timings for the start of orthodontic treatment after periodontal regeneration; note: this study had very few participants in the groups (Table 4).

Papilla presence index improvements (before and after surgical‐orthodontic treatment) were evaluated only in two studies (Cardaropoli *et al*., 2004; Ghezzi *et al*., 2008).

### Tipping, uprighting, intrusion, and extrusion

One of the 14 studies (Eliasson *et al*., 1982) described periodontal changes when treating patients with removable orthodontic appliances and tipping movement. The remaining 13 studies used fixed orthodontic appliances; 11 of the 14 studies investigated periodontal and orthodontic treatment of anterior teeth for pathologic migration, spacing, and marginal bone loss. Intrusion was the most common orthodontic movement (investigated in eight of the 14 studies).

One study evaluated periodontal changes when uprighting molars, and one study evaluated the impact of extrusion of premolar or molar teeth on the periodontal support.

Significant improvement in periodontal status was found in 11 of the 14 studies. Two studies (Eliasson *et al*., 1982; Artun and Urbye, 1988) reported deterioration and improvement of periodontal status; these two studies did not involve periodontal surgery before orthodontic treatment.

In a molar uprighting study (Burch *et al*., 1992), 35% of molars with increased PPD after orthodontic treatment were found, and 60% showed an increase in distance between bone crest and the cemento‐enamel junction at the mesial surface.

### Assessment of the studies

Table 5 summarizes the methodological quality of the 13 clinical studies. The inter‐examiner agreement on each aspect of the Newcastle–Ottawa scale was reached via consensus. The present review observed a consistent finding in eight of the 13 studies, namely, *absence of control groups* (not periodontally involved adults). The Boyd *et al*. (1989) study had a control group that consisted of adolescents, but the study could not be considered *for comparability*. All 13 studies were judged to have low methodological quality.

The RCT study (Ogihara and Wang, 2010) implemented a parallel prospective clinical trial. All patients received initial surgery that applied combined reconstructive approaches using enamel matrix derivative and demineralized freeze‐dried bone allograft on premolar or molar teeth with two‐wall or three‐wall infrabony defects. Following this, patients were assigned to a segmented orthodontic treatment group or no orthodontic treatment group. Teeth in the orthodontic treatment group had extensive subgingival caries and needed crown placement. No mention was made to describe the non‐orthodontic group. The authors did not describe randomization. This was assessed as being inadequate. Allocation concealment (masking of patients and clinicians) was not reported. The authors reported the same number of patients (*n* = 47) had started and had completed the study; no patient was lost. No data were presented regarding adverse effects such as root resorption.

Due to the level of heterogeneity of methodology of the included studies, the reviewers found it impossible to run a meta‐analysis.

## Discussion

This review was limited to periodontal changes when treating patients with chronic periodontal disease. Previous periodontal studies reported that elimination of occlusal traumatic forces improves periodontal tissue healing after periodontal therapy (Burgett *et al*., 1992; McGuire and Nunn, 1996a, 1996b; Harrel and Nunn, 2001a, 2001b). Orthodontic treatment is one modality used to correct traumatic occlusal contacts and to reestablish function and esthetics. Neustadt (1930) and Dummett (1951) recommended orthodontic corrective treatment to eliminate pathologic migration of teeth when managing patients with periodontal disease. Scopp and Bien (1952) reported osseous changes after a tooth extrusion or intrusion, and these changes were related to periodontal disease treatment.

Despite interest in orthodontic treatment on periodontally compromised patients, no studies report rigorous scientific evidence that supports such treatment (Tables 4 and 5).

The selected studies mainly evaluated PPD changes of maxillary anterior teeth. Two studies (Eliasson *et al*., 1982; Artun and Urbye, 1988) reported both – deterioration and improvement of periodontal status – these two studies did not involve periodontal surgery before orthodontic treatment, and this could influence these results. Nine clinical studies showed significant improvement in the post‐treatment status regarding PPDs and/or CCH; see Table 4. Significant PPD reduction was found in studies where intrusion was used to correct extruded maxillary incisors – when comparing baseline and postsurgical‐orthodontic treatment PPD values. The studies (Cardaropoli *et al*., 2001; Corrente *et al*., 2003; Re *et al*., 2004) used open‐flap surgery before orthodontic treatment and used varying techniques for orthodontic intrusion to correct extruded incisors. All three studies (Cardaropoli *et al*., 2001; Corrente *et al*., 2003; Re *et al*., 2004) were performed by the same research group and included patients with migrated and extruded maxillary incisors with radiological presence of infrabony defects and probing depths ≥6 mm (Table 4). The improvement of PPD was related to intrusion, retrusion, and mesial movement of periodontally stable incisors because of previous flaring and/or pathologic overeruption. The positive changes in CCH and PPD during orthodontic movement showed healing of periodontal tissues. But it still remains questionable if a new connective tissue attachment could be created.

Other studies (Melsen et al.1989, Melsen, 2001) also discussed the impact of intrusion to the attachment level changes. They suggested that the fact of PPD improvement could not imply that a new attachment was created, even if histologic studies on monkeys (Melsen et al.1988) may support the possibility. In addition, in the study by Melsen *et al*. (1989), orthodontically intruded upper anterior teeth had developed pockets of about 3 mm, in all cases localized to the lingual surface. At the same time, the measurement of clinical crown length demonstrated a reduction, which was most pronounced lingually. It seems logical, that during intrusion and retrusion of upper incisors, the remodeling of gingival tissues occurs mostly on the lingual aspect of the tooth. However, the clinical parameters, such as PPD and CCH, do not explain the question about new attachment level.

Root resorption due to orthodontic tooth movement is important to document (Lund *et al*., 2012). But in nine of the 14 studies, root resorption was not measured. In the remaining five studies that investigated changes in root length, root resorption was found in two studies (Artun and Urbye, 1988; Melsen *et al*., 1989), and root resorption was not found in three studies; see Table 4.

Even if the methodological quality of included clinical articles is low, the results of this review suggest important information on data available of orthodontic treatment effect on periodontal tissues. Orthodontic treatment, especially intrusion, may help to preserve or even improve the periodontal tissue support around anterior teeth in chronic periodontitis patients. Oral hygiene has to be maintained after active periodontal treatment. As a consequence of intrusion, root resorption may happen. Guided tissue regeneration combined with orthodontic movement suggests better improvement of vertical bone defects around anterior teeth (Table 4).

Because the selected studies report very little information on methodological quality levels, clinical results should be considered with caution.

Going forward, bias‐protected well‐controlled clinical studies are necessary. They should include clinical examinations that cover oral hygiene and periodontal and radiological parameters measured before, during, and after orthodontic treatment – to clarify the safest, most effective method for managing periodontally compromised dentitions. The present review found one RCT, but this study did not present adequate information about randomization procedure, used only segmented orthodontic treatment, and could not provide scientific evidence to answer the research question.

## Conclusions

No evidence currently exists from controlled studies and RCTs, which show that orthodontic treatment improves or aggravates the status of periodontally compromised dentitions.

The null hypothesis was accepted.

## Conflict of Interest

We declare that we have no conflicts of interest in this study.

## Funding Information

No external funding – except support from the authors' institution – was available for this study.
